# Combined Effects of Parenting in Childhood and Resilience on Work Stress in Nonclinical Adult Workers From the Community

**DOI:** 10.3389/fpsyt.2020.00776

**Published:** 2020-07-31

**Authors:** Hiroto Sameshima, Akiyoshi Shimura, Kotaro Ono, Jiro Masuya, Masahiko Ichiki, Satomi Nakajima, Yuko Odagiri, Shigeru Inoue, Takeshi Inoue

**Affiliations:** ^1^Department of Psychiatry, Tokyo Medical University, Shinjuku-ku, Japan; ^2^Department of Psychiatry, Welfare-Kyusyu Hospital, Kagoshima, Japan; ^3^Faculty of Human Sciences, Musashino University, Nishitokyo-shi, Japan; ^4^Department of Preventive Medicine and Public Health, Tokyo Medical University, Shinjuku-ku, Japan

**Keywords:** perceived parental bonding, parental care, parental overprotection, resilience, work stress, structural equation model

## Abstract

**Background:**

Stress responses induced by job stressors are modified by individual factors. Perceived parental bonding and resilience would play important roles as such individual factors. In this study, we analyzed the mediating roles of resilience on parenting, job stressors, and stress responses among adult workers from the community.

**Methods:**

A total of 528 workers participated in this study after providing written consent, and completed questionnaires on demographic data, as well as Parental Bonding Instrument, Connor-Davidson Resilience Scale, and Brief Job Stress Questionnaire. Associations between perceived parental bonding, resilience, perceived job stressors, and the psychological and physical stress response (PPSR) were analyzed using structural equation modeling.

**Results:**

Structural equation modeling with covariance structure analysis showed that parental overprotection reduced resilience and increased perceived job stressors and PPSR. Resilience and perceived job stressors and their combination mediated the effect of parental overprotection on PPSR. Resilience mediated the effect of parental overprotection on perceived job stressors. Perceived job stressors mediated the effect of resilience on PPSR. Parental care had opposite effects to parental overprotection, but the difference was small.

**Conclusion:**

In this study, we showed that perceived parental bonding affects resilience, and that both factors affect the stress response and perceived job stressors. These findings suggest that parental bonding and resilience are major individual factors affecting work stress, and should be noted when considering industrial hygiene measures for individual workers.

## Introduction

The World Health Organization reported that work stress is caused by a mismatch between a person’s knowledge and skills and the job, unmeaningful tasks, unpleasant duties, too much or too little workload, lack of communication, harassment, and conflicts of work-life balance [1]. Work stress causes poor physical and mental health in workers and challenges the health and performance of organizations (1, 2). Initially, Hans Selye proposed that nonspecific reactions to the strain caused by a stimulation (i.e., a stressor) from the external environment is called stress (3). Therefore, work stressors cause psychological and physical responses (PPSR) (reactions or strains) in individuals, and the above systemic changes are called “work stress” (4). Stressors have been considered as objective stimulations, because early studies by Hans Selye used physical stimulation to animals. However, the situation is different for humans, and human individuals evaluate various stressors subjectively (5). Recently, subjective evaluations have mainly been used for measuring the intensity of stressors (5, 6). The Generic Job Stress Questionnaire developed by the National Institute for Occupational Safety and Health, which is a standard evaluation method of work stress, asks respondents to evaluate the intensity of work stressors subjectively (4).

Subjective characteristics of work stressors suggest that various vulnerabilities of individuals, such as early experiences in childhood, personality traits, and genetic factors, may influence work stressors and subsequent stress responses. Previous studies reported that early experiences in childhood, such as parenting and abuse, neuroticism, and life events, interact with each other and finally increases the onset of major depression or the severity of depressive symptoms (7–10). In these models, early experiences in childhood increased neuroticism and subsequently the negative appraisal of adulthood life events (8, 9). This association between factors may apply to work stress. Recently, our group found that perceived parental bonding increased neuroticism, which is a vulnerability for depression and other psychiatric disorders (7, 11), and subsequently worsened work stressors and stress responses (our unpublished data; under submission). These findings suggested the importance of parenting as an individual factor affecting mental health.

Resilience embodies personal qualities that enable a person to thrive in adverse situations (12). Resilience has the following characteristics: quantifiable, affected by health status, modifiable, and improved with treatment (12). Resilience mediates hopelessness in depression (13), and is reported to reduce psychopathology and enhances well-being in postpartum women and corporate executives (14, 15). Childhood adversity (16) and parenting styles, such as care, overprotection, or abuse in childhood influence resilience. Parental warmth was associated with increased resilience, whereas parental protectiveness was associated with decreased resilience, and there was a statistically significant interaction between severe childhood sexual abuse and parental authoritarianism, such that individuals with a childhood sexual abuse history and higher authoritarianism scores had lower resilience (17). Self-esteem, which is an important factor of resilience (18, 19), is enhanced by care parenting and is reduced by abuse or overprotection (20–22). Resilience has opposite effects to neuroticism (8, 9). Parenting may enhance resilience by developing self-esteem, self-confidence, and optimism, and may impair resilience by causing low self-esteem, anxiety, and pessimism. In contrast to neuroticism, our unpublished data has shown that resilience may improve work stressors and subsequent stress responses. Previous studies reported a buffering effect of resilience on job stressors (23–30). However, to our knowledge, there has been no study to date that analyzed the association between parenting, resilience, and stress responses among general workers. Based on the above background information, in this study, we hypothesized that resilience has a mediating role on the effects of perceived parental bonding, perceived job stressors, and the psychological and physical stress response (PPSR). Therefore, in this study, we aimed to verify this hypothesis in adult workers using structural equation modeling.

## Subjects and Methods

### Subjects

Self-administered questionnaires were distributed to 1,237 nonclinical adult workers recruited by convenience sampling through our acquaintances at Tokyo Medical University, from April 2017 to April 2018. Among them, 597 participants (48.3%) gave informed consent and responded to the questionnaire. After the exclusion of invalid answers, 528 participants (42.7%) comprised 233 men and 295 women (average age: 41.4 ± 11.9 years) were analyzed (**Table 1**). This study was part of a larger study, in which several questionnaires were investigated (31). Regarding employment status, 452 were regular employees, six were contract or commission employees, 52 were temporary or part-time employees, and 15 were other types of employees. Regarding job class, 381 were general class employees, 65 were section manager class employees, and seven were manager level or higher employees. Overtime work (hours per month) was 20 h or less in 351 workers, 21–40 h in 74 workers, 41–60 h in 29 workers, and 61 h or more in 16 workers.

**Table 1:** Characteristics, BJSQ, PBI, and CD-RISC of 528 adult workers and their correlation with PPSR score of BJSQ and effects on the stress response score.

**Characteristic or measure**	**Value (number or mean ± SD)**	**The effect on stress response score**
**Age, years**	41.4 ± 11.9	*r* = −0.083, *p* = 0.057
**Sex (men: women)**	233: 295	Men: 51.4 ± 14.3 vs women: 55.9 ± 14.5, *p* < 0.001 (*t*-test)
**Education, years**	14.7 ± 1.8	*r* = −0.059, *p* = 0.178
**Marital status (married: unmarried)**	346: 178	Married: 52.1 ± 14.2 vs unmarried: 57.5 ± 14.9, *p* < 0.001 (*t*-test)
**Living alone (yes: no)**	105: 423	Yes: 56.2 ± 15.2 vs no: 53.3 ± 14.4, *p* = 0.068 (*t*-test)
**Number of offspring**	1.4 ± 1.3	*r* = −0.062, *p* = 0.155
**Comorbidity of physical disease (yes: no)**	104: 424	Yes: 54.7 ± 14.6 vs no: 53.7 ± 14.6, *p* = 0.554 (*t*-test)
**Comorbidity of psychiatric disease (yes: no)**	22: 496	Yes: 67.9 ± 15.5 vs no: 53.3 ± 14.3, *p* < 0.001 (*t*-test)
**First-degree relative with psychiatric disease (yes: no)**	52: 425	Yes: 55.2 ± 13.7 vs no: 53.6 ± 14.6, *p* = 0.449 (*t*-test)
**Occupational contract (permanent: other)**	452: 73	Permanent: 53.7 ± 14.6 vs other: 55.0 ± 14.7, *p* = 0.494 (*t*-test)
**Job class (manager level or higher: section manager class: non-manager)**	7: 65: 381	Manager level or higher: 41.9 ± 8.6 vs section manager class: 50.7 ± 14.7 vs non-manager; 54.3 ± 14.4, *p* = 0.019 (one-way ANOVA)^#^
**Overtime work hours (/month)**	16.4 ± 15.0	*r* = −0.001, *p* = 0.976
**Discretionary work style (yes: no)**	29: 454	Yes: 45.3 ± 11.2 vs no: 54.4 ± 14.5, *p =* 0.001 (t-test)
**CD-RISC score**	55.0 ± 17.4	*r* = −0.357, *p* < 0.001
**BJSQ**		
**Perceived job stressor**	40.6 ± 6.15	*r* = 0.413, *p* < 0.001
** PPSR**	53.9 ± 14.6	*r* = 1
**PBI**		
** Paternal care**	23.5 ± 8.1	*r* = −0.146, *p* = 0.001
** Maternal care**	28.1 ± 6.9	*r* = −0.171, *p* < 0.001
** Paternal overprotection**	9.7 ± 6.9	*r* = 0.212, *p* < 0.001
** Maternal overprotection**	9.6 ± 6.9	*r* = 0.225, *p* < 0.001

Data are presented as means ± SD or numbers.

r = Pearson correlation coefficient.

BJSQ, Brief Job Stress Questionnaire; CD-RISC, Connor-Davidson Resilience Scale; PBI, Parental Bonding Instrument; PPSR, Psychological and Physical Stress Response.

^#^Post-hoc Bonferroni test did not show any statistical group differences.

Participation in the survey was voluntary and not mandatory, and the subjects provided their written informed consent to participate in the survey. All of the data were collected anonymously. This study was approved by the ethics committee of Tokyo Medical University (Approval Number: SH3502).

### Questionnaires

#### Parental Bonding Instrument (PBI)

PBI is a retrospective self-administered questionnaire that measures the child-rearing that participants received from their parents, which is evaluated after the participants become adults (32). PBI consists of 25 questions [13 question items of overprotection (0–39 points) and 12 question items of care (0–36 points)], which are each evaluated using a 4-point Likert scale. The total scores of overprotection and care of the father and mother were used for the analysis. The validated Japanese version of the PBI was used in this study (33).

#### Connor-Davidson Resilience Scale (CD-RISC)

Resilience was assessed using the CD-RISC (12), which is a 25-item, 5-point Likert scale assessment of “personal qualities that enable one to thrive in the face of adversity”. Scores range from 0 (not true at all) to 4 (true nearly all the time). The Japanese version of the CD-RISC, which was confirmed for its validity and reliability by Nakajima et al. (34), was used in this study.

#### Brief Job Stress Questionnaire (BJSQ)

BJSQ is a 57-item multidimensional job stress questionnaire that is used to measure stress response (35, 36). BJSQ is widely used as the recommended protocol of the Stress Check Program in Japan (37, 38). BJSQ includes perceived job stressors (17 items, e.g., quantitative job overload, qualitative job overload, and job control), stress response (29 items, e.g., psychological stress response and physical stress response), social factors (9 items, e.g., family support and colleague support), and satisfaction (2 items). This study analyzed two subscores of perceived job stressors and psychological and physical stress response (PPSR). The higher the score of each subscale, the higher the stress.

### Structural Equation Model

**Figure 1:**
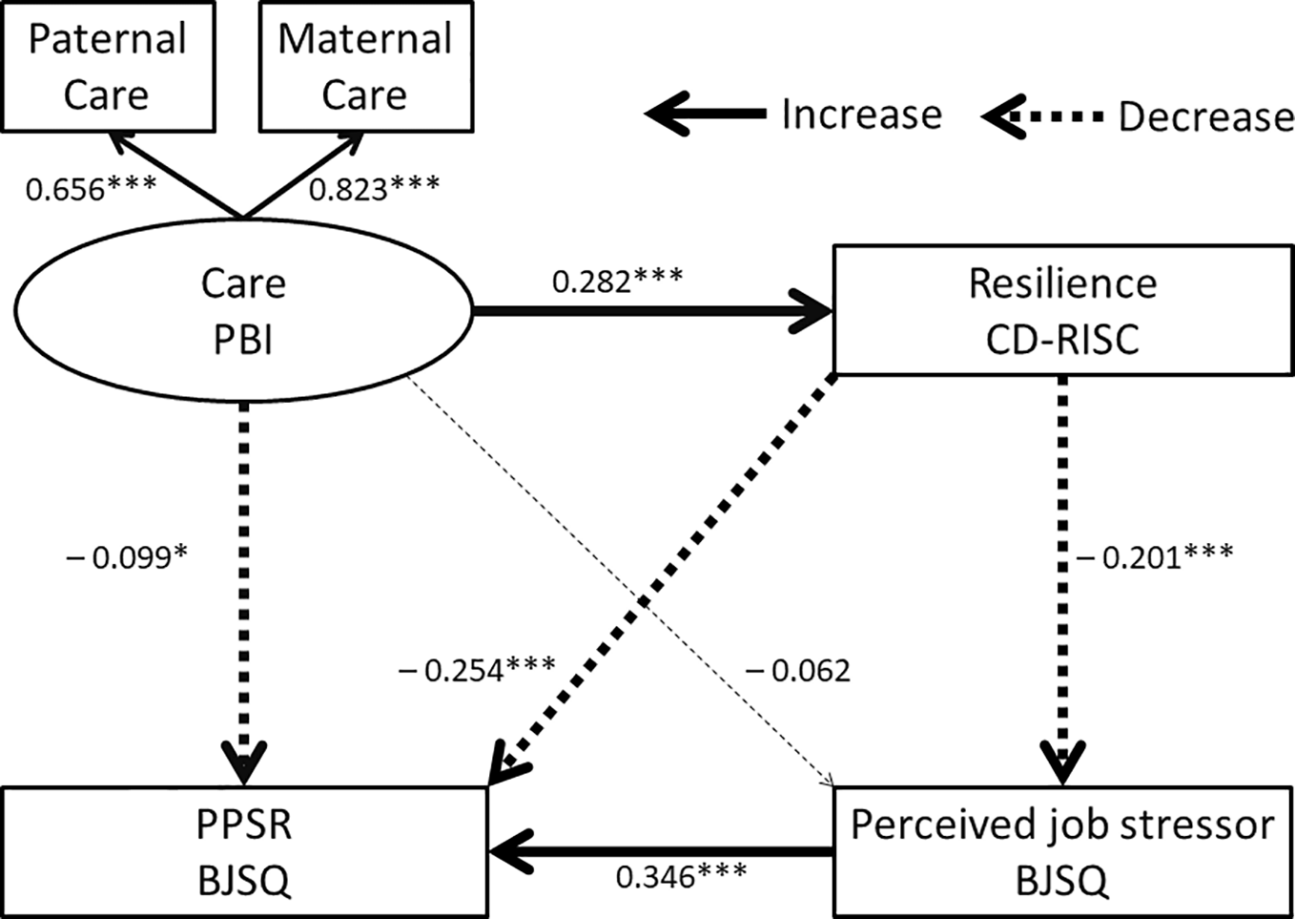
Structural equation model of parental care (PBI), resilience (CD-RISC), perceived job stressors (BJSQ), and PPSR (BJSQ). Solid arrows indicate increased effects, dotted arrows indicate decreased effects, and a thin dotted line indicates a nonsignificant effect. Coefficients beside the lines are standardized. The latent variable “care” consists of paternal and maternal care. *p < 0.05, ***p < 0.001.

**Figure 2:**
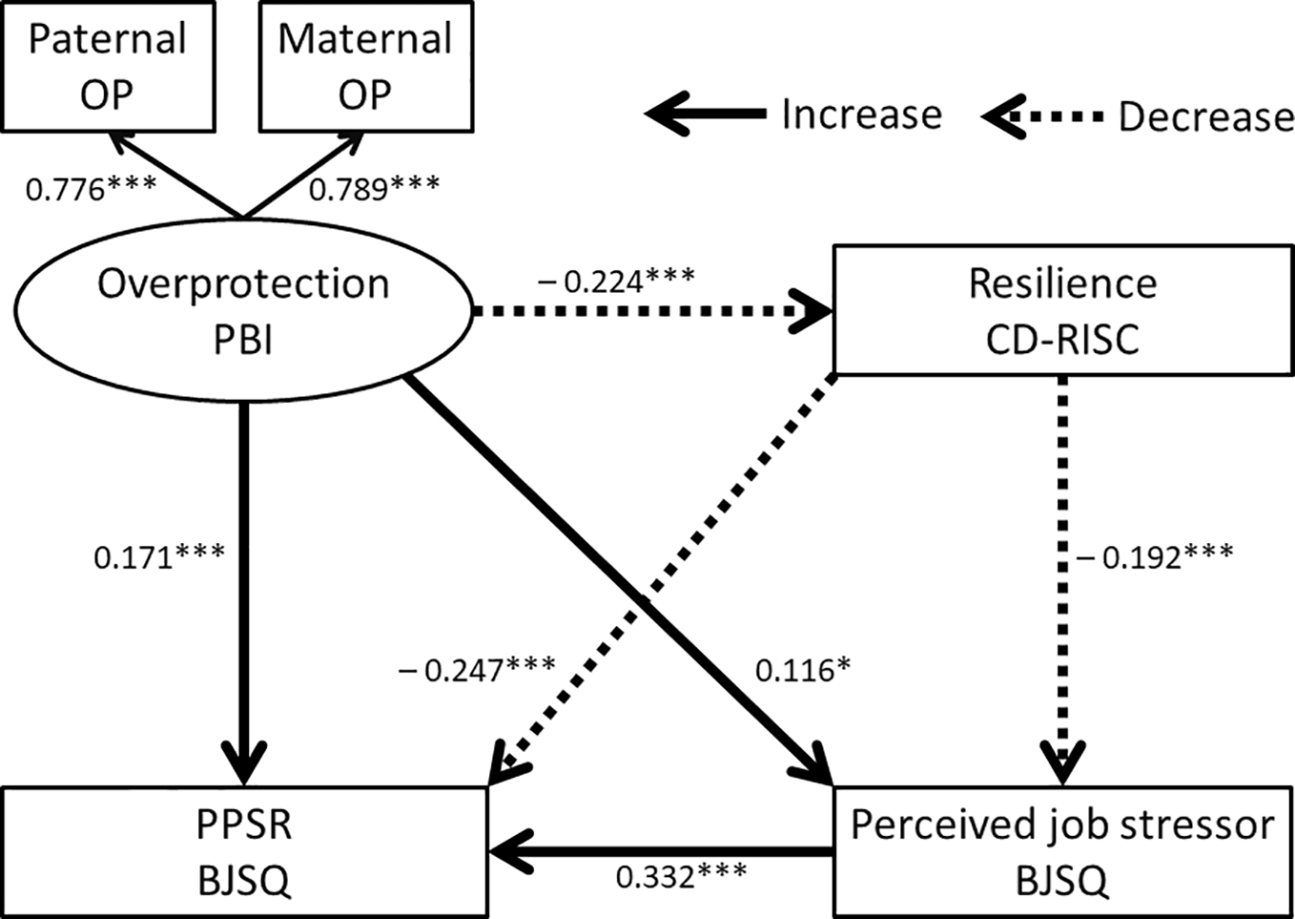
Structural equation model of parental overprotection (PBI), resilience (CD-RISC), perceived job stressors (BJSQ), and PPSR (BJSQ). Solid arrows indicate increased effects and dotted arrows indicate decreased effects. Coefficients beside the lines are standardized. The latent variable “overprotection” consists of both paternal and maternal overprotection (OP). PBI, Parental Bonding Instrument; CD-RISC, Connor-Davidson Resilience Scale; BJSQ, Brief Job Stress Questionnaire. *p < 0.05, ***p < 0.001.

To evaluate the mediating role of resilience on the effects of parental bonding, perceived job stressors, and the PPSR, a structural equation model was built using the scores of the PBI, the CD-RISC, perceived job stressors of the BJSQ, and the PPSR of the BJSQ. According to the two dimensions of PBI, the latent variable of care and overprotection were composed and examined by the two models separately (**Figures 1** and **2**).

### Statistical Analysis

IBM SPSS Statistics Version 25 was used to calculate the Pearson correlation coefficient, and to perform the *t*-test and analysis of variance followed by the Bonferroni test. Mplus version 8.0 (Muthén & Muthén) was used to build the structural equation model with the robust maximum likelihood estimation method. In this study, a comparative fit index (CFI) of greater than 0.97 and the root mean square error approximation (RMSEA) of less 0.05 was considered to indicate a “good fit” (39). All coefficients of the covariance structure analysis were standardized.

## Results

### Demographic and Questionnaire Data of the Subjects

**Table 1** shows the demographic and questionnaire data of 528 adult workers. Sex and marital status were associated with PPSR scores; i.e., women and unmarried subjects showed higher PPSR scores. Other demographic variables were not associated with PPSR scores. Occupational contract, job class, and overtime work hours of participants were not associated with PPSR scores significantly, whereas a discretionary work style showed the lower score of PPSR.

CD-RISC scores were negatively correlated with PPSR scores, and perceived job stressors on the BJSQ were positively correlated with PPSR scores. Parental care and overprotection on the PBI were negatively and positively correlated with PPSR scores, respectively.

### Structural Equation Model

Univariate analyses showed that perceived parental bonding (PBI), resilience (CD-RISC), and perceived job stressors (BJSQ) were correlated with PPSR scores (BJSQ). Based on these results, a hypothesis was built that resilience acts as a mediator of the effects of perceived parental bonding on perceived job stressors and the PPSR. Two structural equation models were built for parental care and overprotection of the PBI as latent variables.

Model 1 for the latent variable of “parental overprotection” is shown in **Figure 2** and the results are also shown in **Table 2**. The fit indices of this model indicated a good fit (RMSEA = 0.012 and CFI = 1.000). The R2 for PPSR was 0.273, indicating that this model explains 27.3% of the variability in the PPSR scores. Paternal overprotection and maternal overprotection contributed to the latent variable of “overprotection” to the same degree, as shown in **Figure 2**. Parental overprotection in childhood directly increased perceived job stressors and PPSR, and directly reduced resilience. Resilience directly reduced perceived job stressors and PPSR.

**Table 2:** Standardized path coefficients between each variable of parental overprotection.

	Direct effect on		
From	Resilience	Job stressor	PPSR(BJSQ)
**Overprotection (PBI)**	−0.224***	0.116*	0.171***
**Resilience (CD-RISC)**		–0.192***	–0.247***
**Job stressor (BJSQ)**			0.332***
	**Indirect effect on**		
		**Job stressor**	**PPSR** **(BJSQ)**
**Overprotection (PBI)**	Via Resilience	0.043**	0.055***
	Via Job stressor		0.039*
	Via Resilience + job stressor		0.014**
	Total indirect effect		0.108***
**Resilience (CD-RISC)**	Via Job stressor		–0.064***
	**Total effect on**		
		**Job stressor**	**PPSR**
**Overprotection (PBI)**		0.160**	0.279***
**Resilience (CD-RISC)**		–	–0.310***

*p < 0.05, **p < 0.01, ***p < 0.001.

PPSR, Psychological and Physical Stress Response; BJSQ, Brief Job Stress Questionnaire; PBI, Parental Bonding Instrument; CD-RISC, Connor-Davidson Resilience Scale; BJSQ, Brief Job Stress Questionnaire.

Parental overprotection in childhood indirectly enhanced perceived job stressors and PPSR through reduced resilience (**Table 2**), and parental overprotection indirectly enhanced PPSR through increased perceived job stressors. Parental overprotection also indirectly enhanced PPSR through combined pathways involving both resilience and perceived job stressors. Resilience indirectly decreased PPSR through decreased perceived job stressors.

Model 2 for the latent variable of “parental care” is shown in **Figure 1**, and the results are also shown in **Table 3**. The fit indices of this model indicated a good fit (RMSEA = 0.000 and CFI = 1.000). The R2 for PPSR was 0.255, indicating that this model explains 25.5% of the variability in PPSR scores. Maternal care contributed to the latent variable of “care” in **Figure 1** more than paternal care. Parental care in childhood directly decreased PPSR and directly increased resilience. The effect of parental care on perceived job stressors was not statistically significant. Resilience directly reduced perceived job stressors and PPSR.

Parental care in childhood indirectly decreased perceived job stressors and PPSR through increased resilience (**Table 3**). The indirect effect of parental care on PPSR through perceived job stressors was not statistically significant. Parental care also indirectly decreased PPSR through combined pathways involving both resilience and perceived job stressors. Resilience indirectly decreased PPSR through decreased perceived job stressors.

**Table 3:** Standardized path coefficients between each variable of parental care.

	Direct effect on		
From	Resilience	Job stressor	PPSR(BJSQ)
**Care (PBI)**	0.282***	−0.062	−0.099*
**Resilience (CD-RISC)**		–0.201***	–0.254***
**Job stressor (BJSQ)**			0.346***
	**Indirect effect on**		
		**Job stressor**	**PPSR** **(BJSQ)**
**Care (PBI)**	via Resilience	−0.057**	−0.072***
	via Job stressor		−0.022
	via Resilience + job stressor		−0.020**
	Total indirect effect		−0.113***
**Resilience (CD-RISC)**	via Job stressor		–0.070***
	**Total effect on**		
		**Job stressor**	**PPSR**
**Care (PBI)**		−0.119	−0.212***
**Resilience (CD-RISC)**		–	–0.323***

*p < 0.05, **p < 0.01, ***p < 0.001.

BJSQ, Brief Job Stress Questionnaire; PBI, Parental Bonding Instrument; CD-RISC, Connor-Davidson Resilience Scale; BJSQ, Brief Job Stress Questionnaire; PPSR, Psychological and Physical Stress Response.

To examine the effect of sex on the models, the additional structural equation models, including sex as an observed variable were built, and the significances of direct and indirect coefficients of paths were not different from Model 1 and 2 (data not shown).

## Discussion

The main finding of this study is that perceived parental bonding, i.e., the quality of parenting received in childhood, influences a subject’s resilience in adulthood and subsequently influences work stress, in which perceived job stressors cause PPSR in adult workers in the community. Structural equation modeling indicates that resilience is a mediator of the effects of perceived parental bonding on perceived job stressors and PPSR. Therefore, when the job stress of workers is evaluated and intervention is performed, parenting and resilience should be considered as individual factors in the care of industrial mental health in workplaces.

Resilience is now receiving increasing interest in the fields of policy and practice in health, because “deficit” models of illness provide dissatisfaction to subjects in their recovery and health (40). Resilience reflects the ability of a person to maintain a stable physical and psychological equilibrium, which is aimed toward maintaining lifelong health and well-being and against trauma and loss (41). High resilience is reportedly associated with less depression, less anxiety, and lower stress (14, 15, 42). Low resilience was observed in patients with post-traumatic stress disorder, who were exposed to actual or the threat of death, serious injury, or sexual violence (12, 43, 44). Interestingly, resilience as evaluated by CD-RISC is modifiable and can improve with pharmacological treatment, and predicts treatment responses (12, 45). The above facts of resilience suggest that resilience may affect work stress, including job stressors and subsequent stress responses. Previous studies reported the buffering effect of resilience on job stressors (23–29). However, to the best of our knowledge, no studies to date have investigated the mechanism of the association among parenting, resilience, job stressors, and the stress response, and our present study is the first to show the associations among these factors. Resilience directly decreased perceived job stressors and decreased PPSR directly and indirectly through its effects on perceived job stressors. The above definition of resilience explains its buffering effect on work stress, and the promotion of resilience is hence a target for industrial mental health measures in the future.

As expected from the results of previous studies showing that early experiences in childhood affect personality traits (6, 8, 9, 20, 46–51), parental care in childhood increased resilience and parental overprotection decreased it in this study. Consistent with our results, Lind et al. reported the same findings, and also demonstrated that the interaction of childhood sexual abuse with high parental authoritarianism affected resilience (17). Our results replicated the results of Lind et al. and further demonstrated that resilience mediated the effect of perceived parental bonding on work stress. Such a mediating effect of resilience has never been reported to date, but it is plausible considering that resilience is a type of positive personality trait; mediating effects between early experiences and life events or psychopathological symptoms have been shown for various personality traits (6, 8, 9, 48–51). A previous systematic review showed that amenable resilience-enhancing factors moderate and/or mediate the association between childhood adversity and mental health in young people (16). In addition, resilience mediates the association between interpersonal risk factors (i.e., stressors) and hopelessness (13). In other aspects, resilience is reportedly a moderator between trauma and PTSD symptoms, and between stressful life events and sleep quality (52, 53). These previous studies support our present findings of the mediating effects of resilience on stress and mental health. Future studies analyzing the mediating effects of resilience as well as its moderating effects should be analyzed and verified in a prospective study.

The model of job stress and health proposed by the National Institute for Occupational Safety and Health demonstrates that job stressors are located most upstream in its model, and individual factors are treated as moderators between job stressors and stress reactions leading to illness (4). Individual workers have long life histories, which may include early positive or negative experiences in childhood, and these early experiences influence personality traits, such as neuroticism and resilience. As mentioned in *Introduction*, the subjective nature of job stressors indicates that the intensity of stressors depends on individual personality traits, i.e., an individual’s sensitivity to stressors. Furthermore, neuroticism and resilience may be altered by childhood experiences, life events, and treatment (12, 41, 54, 55). Therefore, resilience can be not only a moderator of stress but also a mediator of stress, as shown by the results of this study. Finally, the significant mediation effect of the combination of resilience and perceived job stressors between perceived parental bonding and PPSR supports the above idea. Therefore, life history and personality traits influence the intensity of job stressors and subsequent stress responses leading to illness.

There are some limitations to this study. The retrospective evaluation of perceived parental bonding may be affected by recall bias and the psychopathology of participants. PPSR scores were not limited to work stress because of the method of questioning, but the relatively high path coefficients of the pathway from perceived job stressors to PPSR, and the significant indirect effects of perceived parental bonding and resilience on PPSR through perceived job stressors guarantee this link to some extent. Psychiatric comorbidity also would affect these variables. However, since the number of the participants was limited, the effect was not examined enough. In addition, the cross-sectional design of this study did not enable us to conclude whether there was a causal association between the factors.

## Conclusions

This study showed that perceived parental bonding affects resilience, and both factors affect the effects of perceived job stressors on the stress response. Structural equation modeling indicates that there are significant indirect effects of perceived parental bonding and resilience on perceived job stressors and the stress response. These findings suggest that parental bonding and resilience could be important individual factors affect work stress, and should be considered when designing industrial hygiene measures for individual workers.

## Data Availability Statement

The raw data supporting the conclusions of this article will be made available by the authors, without undue reservation.

## Ethics Statement

The studies involving human participants were reviewed and approved by Ethics Committee of Tokyo Medical University. The patients/participants provided their written informed consent to participate in this study.

## Author Contributions

HS, AS, and TI designed the study, collected the data, and wrote the protocol. TI supervised the whole of research project. All authors contributed to the article and approved the submitted version.

## Conflict of Interest

AS has received fees from Meiji Seika Pharma, Yoshitomi Yakuhin, Tanabe Mitsubishi Pharma, and Eisai outside of the submitted work and is a stockholder of Children and Future Co., Ltd. JM has received personal compensation from Otsuka Pharmaceutical, Eli Lilly, Astellas, and Meiji Yasuda Mental Health Foundation, and grants from Pfizer. TI has received personal fees from Mochida Pharmaceutical, Takeda Pharmaceutical, Eli Lilly, Janssen Pharmaceutical, MSD, and Taisho Toyama Pharmaceutical, Yoshitomiyakuhin, and Daiichi Sankyo; grants from Shionogi, Astellas, Tsumura, and Eisai; and grants and personal fees from Otsuka Pharmaceutical, Dainippon Sumitomo Pharma, Mitsubishi Tanabe Pharma, Kyowa Pharmaceutical Industry, Pfizer, Novartis Pharma, and Meiji Seika Pharma; and is a member of the advisory boards of Pfizer, Novartis Pharma, and Mitsubishi Tanabe Pharma.

The remaining authors declare that the research was conducted in the absence of any commercial or financial relationships that could be construed as a potential conflict of interest.
