# Corrigendum: Combined Effects of Parenting in Childhood and Resilience on Work Stress in Nonclinical Adult Workers From the Community

**DOI:** 10.3389/fpsyt.2021.742812

**Published:** 2021-09-16

**Authors:** Hiroto Sameshima, Akiyoshi Shimura, Kotaro Ono, Jiro Masuya, Masahiko Ichiki, Satomi Nakajima, Yuko Odagiri, Shigeru Inoue, Takeshi Inoue

**Affiliations:** ^1^Department of Psychiatry, Tokyo Medical University, Shinjuku-Ku, Japan; ^2^Department of Psychiatry, Welfare-Kyusyu Hospital, Kagoshima, Japan; ^3^Faculty of Human Sciences, Musashino University, Nishitokyo-Shi, Japan; ^4^Department of Preventive Medicine and Public Health, Tokyo Medical University, Shinjuku-ku, Japan

**Keywords:** perceived parental bonding, parental care, parental overprotection, resilience, work stress, structural equation model

In the original article, the legends for **Figures 1** and **2** were swapped.

**Figure 1**. Structural equation model of parental care (PBI), resilience (CD-RISC), perceived job stressors (BJSQ), and PPSR (BJSQ). Solid arrows indicate increased effects, dotted arrows indicate decreased effects, and a thin dotted line indicates a nonsignificant effect. Coefficients beside the lines are standardized. The latent variable “care” consists of paternal and maternal care. ^*^p < 0.05, ^***^p < 0.001.

**Figure 2**. Structural equation model of parental overprotection (PBI), resilience (CD-RISC), perceived job stressors (BJSQ), and PPSR (BJSQ). Solid arrows indicate increased effects and dotted arrows indicate decreased effects. Coefficients beside the lines are standardized. The latent variable “overprotection” consists of both paternal and maternal overprotection (OP). PBI, Parental Bonding Instrument; CD-RISC, Connor-Davidson Resilience Scale; BJSQ, Brief Job Stress Questionnaire. ^*^p < 0.05, ^***^p < 0.001.

In the original article, there was an error. The quoted variables from the figure were wrong in the **Results** section. A correction has been made to **Results**, **Structural Equation Model**, **paragraphs 2** and **4**:

Model 1 for the latent variable of “parental overprotection” is shown in **Figure 2** and the results are also shown in Table 2. The fit indices of this model indicated a good fit (RMSEA = 0.012 and CFI = 1.000). The *R*^2^ for PPSR was 0.273, indicating that this model explains 27.3% of the variability in the PPSR scores. Paternal overprotection and maternal overprotection contributed to the latent variable of “overprotection” to the same degree, as shown in **Figure 2**. Parental overprotection in childhood directly increased perceived job stressors and PPSR, and directly reduced resilience. Resilience directly reduced perceived job stressors and PPSR.

Model 2 for the latent variable of “parental care” is shown in **Figure 1**, and the results are also shown in Table 3. The fit indices of this model indicated a good fit (RMSEA = 0.000 and CFI = 1.000). The *R*^2^ for PPSR was 0.255, indicating that this model explains 25.5% of the variability in PPSR scores. Maternal care contributed to the latent variable of “care” in **Figure 1** more than paternal care. Parental care in childhood directly decreased PPSR and directly increased resilience. The effect of parental care on perceived job stressors was not statistically significant. Resilience directly reduced perceived job stressors and PPSR.

The authors apologize for these errors and state that this does not change the scientific conclusions of the article in any way. The original article has been updated.

## Publisher's Note

All claims expressed in this article are solely those of the authors and do not necessarily represent those of their affiliated organizations, or those of the publisher, the editors and the reviewers. Any product that may be evaluated in this article, or claim that may be made by its manufacturer, is not guaranteed or endorsed by the publisher.

